# Four-Year Experience of HIV Pre-exposure Prophylaxis (PrEP) from an Italian Multicentre Cohort: Incidence of Sexually Transmitted Infections and Renal Toxicity

**DOI:** 10.1007/s10461-025-04736-5

**Published:** 2025-05-22

**Authors:** Marianna Menozzi, Maria Daria Di Trapani, Giulia Jole Burastero, Sara Volpi, Gianluca Cuomo, Maria Mazzitelli, Francesco Barbaro, Laura Labate, Anna Maria Cattelan, Antonio Di Biagio, Cristina Mussini

**Affiliations:** 1 Clinic of Infectious Diseases, University Hospital of Modena, University of Modena and Reggio Emilia, Modena, Italy; 2 Infectious and Tropical Diseases Unit, Padua University Hospital, Padua, Italy; 3 Infectious Disease Clinic, Department of Health Sciences IRCCS Ospedale Policlinico San Martino, University of Genova, Genova, Italy

**Keywords:** PrEP, STI, HIV, Nephrotoxicity, Prevention, AIDS, Tenofovir/Emtricitabine, PrEP, ITS, VIH, Nefrotoxicidad, Prevención, SIDA, Tenofovir/Emtricitabina

## Abstract

Pre-Exposure Prophylaxis (PrEP) with Tenofovir-Disoproxil/Emtricitabine (TDF/FTC) is efficacious for HIV prevention. PrEP users might be more exposed to other sexually transmitted infections (STIs) and toxicities. Our aim was to evaluate the trend of STIs and toxicities in an Italian PrEP cohort. A retrospective multicentre cohort study (Modena, Genova and Padova) including TDF/FTC-PrEP users followed from Jan-2019 to Jul-2022. Data collection included demographics, toxicities and STIs detection: *N.gonorrhoeae* (NG), *C.trachomatis* (CT), *M.genitalium* (MG), *T.vaginalis* (TV), *T.pallidum* (TP), HAV, HBV and HCV. Two-hundred-forty-four persons included: 97% males; median age 39 years (IQR 33–47). A nearly incremental trend was recorded in NG and MG incidences, especially after 2020: from 5.7% to 2.3% in 2020, to 10.3% and 6.5% in 2021 and to 9.6% and 4.8% in the first 7 months of 2022. CT and TP presented a variable trend, while only two TV diagnoses were done. The test for trend for ordered groups across years showed no statistical significance for all the STIs and the annual proportions of subjects with multiple STIs varied. At logistic regression, only history of STIs was associated to risk of new STIs. Twenty cases of nephrotoxicity were recorded, leading to PrEP interruption in 1 case only. Concluding, STIs incidence and nephrotoxicity in our cohort were consistent with other data from literature. In 2020 we observed a lower STIs incidence, probably as consequence of COVID-19 restrictions. An incremental trend could be hypothesized regarding NG, MG and CT incidence. Thus, we suggest STIs monitoring, prophylaxis and treatment to contain their spread among PrEP users.

## Resumen

Tenofovir-disoproxil/emtricitabina (TDF/FTC) como profilaxis preexposición (PrEP) es eficaz para prevenir el VIH. PrEP usuarios podría aumentar la exposición a otras infecciones de transmisión sexual (ITS) y toxicidades. Este estudio multicéntrico retrospectivo (Módena, Génova, Padua) incluyó 244 usuarios de PrEP (97% varones, edad mediana 39 años) desde enero-2019 hasta julio-2022, con el objetivo de evaluar la tendencia de ITS y toxicidades. Tras 2020, la incidencia de *N.gonorrhoeae* (NG) y *M.genitalium* (MG) mostró una tendencia casi incremental, y la proporción de sujetos con múltiples ITS varió anualmente. El antecedente de ITS se asoció con riesgo de nuevas infecciones. Veinte casos de nefrotoxicidad registrados, con una interrupción de la PrEP. Concluyendo, la incidencia de ITS y toxicidad fue coherente con datos previos. En 2020 se observó una menor incidencia de ITS, posiblemente debido a las restricciones por COVID-19. Se sugiere una tendencia creciente en ITS, lo que refuerza monitorización y prevención en usuarios de PrEP.

## Introduction

The Pre-Exposure Prophylaxis (PrEP) is an efficacious strategy to prevent HIV transmission recommended by national and international guidelines throughout the continents [1, 2]. To date it represents a cornerstone in terms of reduction of new HIV diagnoses and against the HIV pandemic.

The oral intake of tenofovir disoproxil fumarate/emtricitabine (TDF/FTC), by a “daily” or “on demand” approach, has been demonstrated to be safe and efficacious in people considered at risk of acquiring HIV [3, 4, 5]. For this reason, the CDC now recommends discussing PrEP with all sexually active adults and adolescents and prescribing PrEP to people who request it, even if they do not report sexual or drug-injection practices that may put them at risk of acquiring HIV [6].

PrEP should be considered as a complementary method of prevention, together with the use of condoms, an adequate sexual education and should be routinely integrated in all sexual health-care systems. However, concerns still remain about the phenomenon of risk compensation, i.e., a reduced sexual risk perception derived from the awareness of PrEP efficacy against HIV transmission, which can lead to an increase in Sexually Transmitted Infections (STIs) spread [7, 8].

Literature is not unanimous about the role of PrEP as driver of possible change in risky sexual behaviors, leading instead to an increased risk of STIs acquisition [9, 10, 11]. Indeed, bacterial STI rate is constantly rising worldwide including Italy, especially in men who have sex with men (MSM) [12, 13]. Considering this phenomenon, some randomized clinical trials were performed to assess the effectiveness and safety of doxycycline, as post-exposure prophylaxis (doxy-PEP) to prevent STI transmission in PrEP users with successful results [14, 15, 16].

Another matter of concern about the use of PrEP is represented by long-term potential toxicity of TDF, especially on kidney function. Kidney injury caused by TDF, manifesting as acute or chronic damage, as well as Fanconi syndrome, is well known and documented in people living with HIV (PLWH) using TDF containing regimens [17]. Some studies investigated the impact of PrEP on serum creatinine levels and eGFR evolution, showing a worsening of kidney function, after a variable period of PrEP intake. These variations seem to be correlated with an older age and a lower initial eGFR [18, 19]. Even if complete functional recovery has been shown after drug discontinuation, irreversible renal impairment in a long-term daily use cannot be excluded [20, 21].

In this scenario of rising STI rate within the MSM population, being the most involved in PrEP prescription, the aim of our study was to describe the PrEP experience of three Italian centres, focusing on the population characteristics and the rate of STIs. A secondary objective has been to describe the possible impact of PrEP on renal function, being the major risk of TDF administration.

## Methods

We conducted a retrospective observational multicenter study including all PrEP users followed in the dedicated services of three Italian Infectious Disease departments (University Hospital of Modena, San Martino Hospital of Genoa, and Padua University Hospital). Data were collected quarterly between January 2019 and July 2022. PrEP was dispensed either “daily” or “on demand”, according to risk behavior temporality disclosed by users. Demographic information (age, sex, nationality), reason of PrEP begin, use of condom and sexual habits, were recorded at baseline and updated at each follow-up examination. Reason for PrEP begin were self-reported and could be multiple for each individual. We grouped reason for PrEP begin in two groups according to the self-awareness of the risk or to the external suggestion by ID specialist (due to previous STI diagnosis or Post Exposure Prophylaxis [PEP] prescription).

The first assessment was scheduled after one month from TDF/FTC prescription; then a quarterly monitoring was provided through clinical evaluation, blood check and an STIs screening including HIV, *Treponema pallidum* (TP), *Neisseria gonorrhoeae* (NG), *Chlamydia trachomatis* (CT), *Mycoplasma genitalium* (MG), *Trichomonas vaginalis* (TV), hepatitis B virus (HBV), hepatitis C virus (HCV) and hepatitis A virus (HAV) screening. Infections due to NG, CT, MG, and TV were detected with a polymerase chain reaction (PCR) in urine, rectal and pharyngeal samples. TP was evaluated by a two steps assay based on screening with TP particular agglutination (TPPA) followed, if positive, by standard VDRL. HIV was assessed through a fourth-generation test and serologies were performed for HBV, HCV and HAV.

STI incidence was defined as number of new diagnosed infections per total number of tests performed by users per each year of follow-up.

Among blood tests, we checked glomerular filtration rate, serum creatinine, phosphoremia in order to assess renal function, and serum transaminase (ALT). We monitored all the adverse events, including renal, liver, and gastrointestinal toxicity, any reason of PrEP discontinuation with its rates. Nephrotoxicity was defined as an increase of at least 20% in creatinine serum level from baseline to last follow-up available. Liver toxicity was defined as ALT elevation > 3 times above the upper normal reference value. Gastrointestinal toxicity included nausea, vomiting, diarrhoea, and gastric pain.

A descriptive analysis of the population demographic characteristics was performed using median (Inter-Quartile Range, IQR) and number (frequency) for continuous and categorical variables respectively. To better describe our population, we divided it by age group at time of PrEP initiation (< 30 year-old, 31–50 year-old and > 50 year-old), doing comparisons with Mann-Whitney U test, ANOVA and Chi-square test according to type and distribution of variables.

Regarding STI incidence, a test for trend across ordered groups (linear-by-linear trend test) was used to evaluate the presence of incidence variations of the single STIs during years of follow-up, based on the total number of users followed for the specific year.

Logistic univariate and multivariate regression models were created to address factors potentially associated with the risk of STIs. Covariates included were: sex, age at PrEP initiation, nationality, reason of begin, previous STIs diagnosis, PrEP regimen and condom use (assessing condom use as “not known” if not reported).

The sub-analysis on nephrotoxicity, defined as described above, was performed initially including subjects with full renal function data: a description of the cases was done about age and type of PrEP administration. Data were fully available (at baseline and last determination) only for a subset of individual, while 22.5% of the renal-function variables were missing. To address this issue, we analysed the dataset and found that baseline and final creatinine values did not differ significantly between missing and non‐missing groups, thus we proceeded with multiple imputation tests for these variables. We used different multiple imputation techniques: Multiple Imputation by Chained Equations (MICE), k‐Nearest Neighbors (kNN) and a “mixed” approach (MICE for continuous variables and kNN for binary variables). We then compared the results using divergence metrics (e.g., KL divergence, Jensen‐Shannon Divergence, Kolmogorov–Smirnov Distance, and Chi-Square Distance for binary data). kNN produced smaller divergences overall, indicating that it better preserved the original distributional properties of both continuous and binary variables. Therefore, we adopted kNN for imputing baseline and final creatinine values. In terms of addressing potential systematic bias, we conducted (1) statistical tests to assess whether those with missing values differ systematically from those with complete data (our “missing at random” checks) and (2) sensitivity analyses by comparing the correlation structures pre‐ and post‐imputation. Correlation matrix results are obtained by using: Spearman correlation (float-float corr), point biserial correlation (float-bin corr) and Cramer’s V coefficient (bin-bin corr). The results showed that the correlation structure in the dataset was well preserved. These steps minimized the risk of biased estimates and helped ensuring our findings remain robust. Hence, despite the 22.5% missing data, using kNN imputation with appropriate follow‐up sensitivity analyses mitigated the impact of missing data on our study results and upheld their credibility.

We then performed a new statistical analysis with imputed data.

All the analyses were performed with Stata Corp ver 15.0; all p-values were 2-tailed and significance level was set < 0.05.

## Results

Over this period, 244 people were included, 97% cisgender men, 1% cisgender women, 1% transgender women; median age was 39 years (IQR 33–47). Table 1 depicts numbers and frequencies of users’ inclusion in the data collection, by year of PrEP initiation and sex.

**Table 1:** Number of people who started PrEP divided by year

Sex, n	Year of PrEP beginning				
	2019	2020	2021	7 months 2022	Overall, n (%)
Cisgender women	0	3	0	1	4 (1)
Cisgender men	50	30	86	72	238 (97)
Transgender women	1	0	1	0	2 (1)
Overall	51	33	87	73	244
Cumulative amount	–	84	171	244	

Table 2 shows population characteristics including demographics, PrEP regimen, starting and interruption reasons, side effects and condom use, divided by age strata. The majority of users were Italian (91.8%). In particular, we recorded that a great proportion of users reported Self-awareness of the risk as main reason to begin PrEP (75%), while less than 20% of users reported to be addressed to PrEP by ID specialist after STIs diagnosis or PEP prescription. Use of condom was intermittent in 69.5% of cases and never used in 11%.

**Table 2:** Study population characteristics by age group

Characteristic	Age group of PrEP initiation				
	≤ 30 years 52 (21.3)	31–50 years 154 (63.1)	> 50 years 38 (15.6)	Overall, *n* = 244	p-value*
*Sex, n (%)*					0.207
Cisgender men	51 (98)	150 (97.4)	37 (97.4)	238 (97.5)	
Cisgender women	0	4 (2.6)	0	4 (1.7)	
Transgender women	1 (2)	0	1 (2.6)	2 (0.8)	
*Nationality, n (%)*					0.004
Italian	42 (80.8)	145 (94.2)	37 (97.4)	224 (91.8)	
Non-Italian	10 (19.2)	9 (5.8)	1 (2.6)	20 (8.20)	
*MSM, n (%)– cisgender males only*					0.928
No	1 (2.5)	2 (1.3)	1 (2.6)	4 (1.7)	
Yes	44 (86.3)	135 (90)	33 (89.2)	212 (89.1)	
Not declared	6 (11.8)	13 (8.7)	3 (8.1)	22 (9.2)	
*Regimen, n (%)*					0.500
On-demand	24 (53.3)	79 (56.8)	29 (63.3)	122 (57)	
Daily	16 (35.6)	41 (29.5)	9 (30)	66 (30.8)	
Both	5 (11.1)	19 (13.7)	2 (6.7)	26 (12.2)	
*Reason of begin, n (%)*					0.392
Self-awareness of risk	36 (69.2)	116 (75.3)	31 (81.6)	183 (75)	
Suggested by ID specialist	10 (19.2)	28 (18.2)	3 (7.9)	41 (16.8)	
Not declared	6 (11.5)	10 (6.5)	4 (10.5)	20 (8.2)	
Interruptions, n (%)	10 (19.2)	20 (12.9)	2 (5.3)	32 (13.1)	0.152
*Interruption reason, n (%) -within interruptions (32 subj)*					0.740
Stable partner	2 (20)	1 (5)	0	3 (9.4)	
Lost to follow-up	7 (70)	10 (50)	2 (100)	19 (59.4)	
Toxicity	0	1 (5)	0	1 (3.1)	
Lockdown	0	4 (20)	0	4 (12.5)	
Parenthood	0	1 (5)	0	1 (3.1)	
Not known	1 (10)	3 (15)	0	4 (12.5)	
*Side effects, n (%)*					0.713
None	41 (78.9)	113 (73.4)	32 (84.2)	186 (76.2)	
Gastrointestinal disturbs	8 (15.4)	28 (18.8)	5 (13.2)	41 (16.8)	
Neprhotoxicity	1 (1.9)	6 (3.9)	0	7 (2.9)	
Hepatic toxicity	0	4 (2.6)	0	4 (1.6)	
Other	1 (1.9)	2 (1.3)	0	3 (1.2)	
Not known	1 (1.9)	1 (0.7)	1 (2.6)	3 (1.2)	
*Previous STI, n (%)*					0.352
None	8 (20)	22 (13.5)	4 (9.8)	34 (13.9)	
Yes	20 (50)	83 (59.9)	27 (65.9)	130 (53.3)	
Not known	12 (30)	58 (35.6)	10 (24.3)	80 (32.8)	
*Condom use, n (%)*					0.090
Never	9 (23.1)	10 (8.2)	2 (6.9)	21 (11)	
Always	5 (12.8)	25 (20.5)	7 (24.1)	37 (19.5)	
Intermittent	25 (64.1)	87 (71.3)	20 (69)	132 (69.5)	
Overall	39	122	29	190	

* Mann-Whitney U test and ANOVA for continuous variables and Chi-square test for categorical variables

Median follow-up duration had been 0.65 years (IQR 0.25–1.7 years), with a maximum follow-up of 3.4 years. Fifty-two and 25 users presented a follow-up above 2 and 3 years, respectively. A total of 98,150 patient-days-follow-up was calculated.

We experienced some loss-to-follow-up, after a median of 0.49 years (IQR 0.21–0.50 years), mainly recorded in the years 2020 and 2021.

PrEP interruption occurred in 32 individuals (13.1%), after a median time of 0.49 years (IQR 0.34–1.83 years). A single user interrupted prophylaxis due to toxicity (1 out of 244 users, 0.41%), in particular the user experienced nephrotoxicity.

Side effects occurred in 55 subjects (22.5%): 41 gastrointestinal disorders (17%), 4 hepatic toxicities (1.7%), 7 nephrotoxicity (2.9%), 3 others (1.2%).

Before PrEP initiation more than half of the population (53%) reported history of STI, although history data were not available for all the users.

### Sexually Transmitted Infection Analysis

**Fig. 1:**
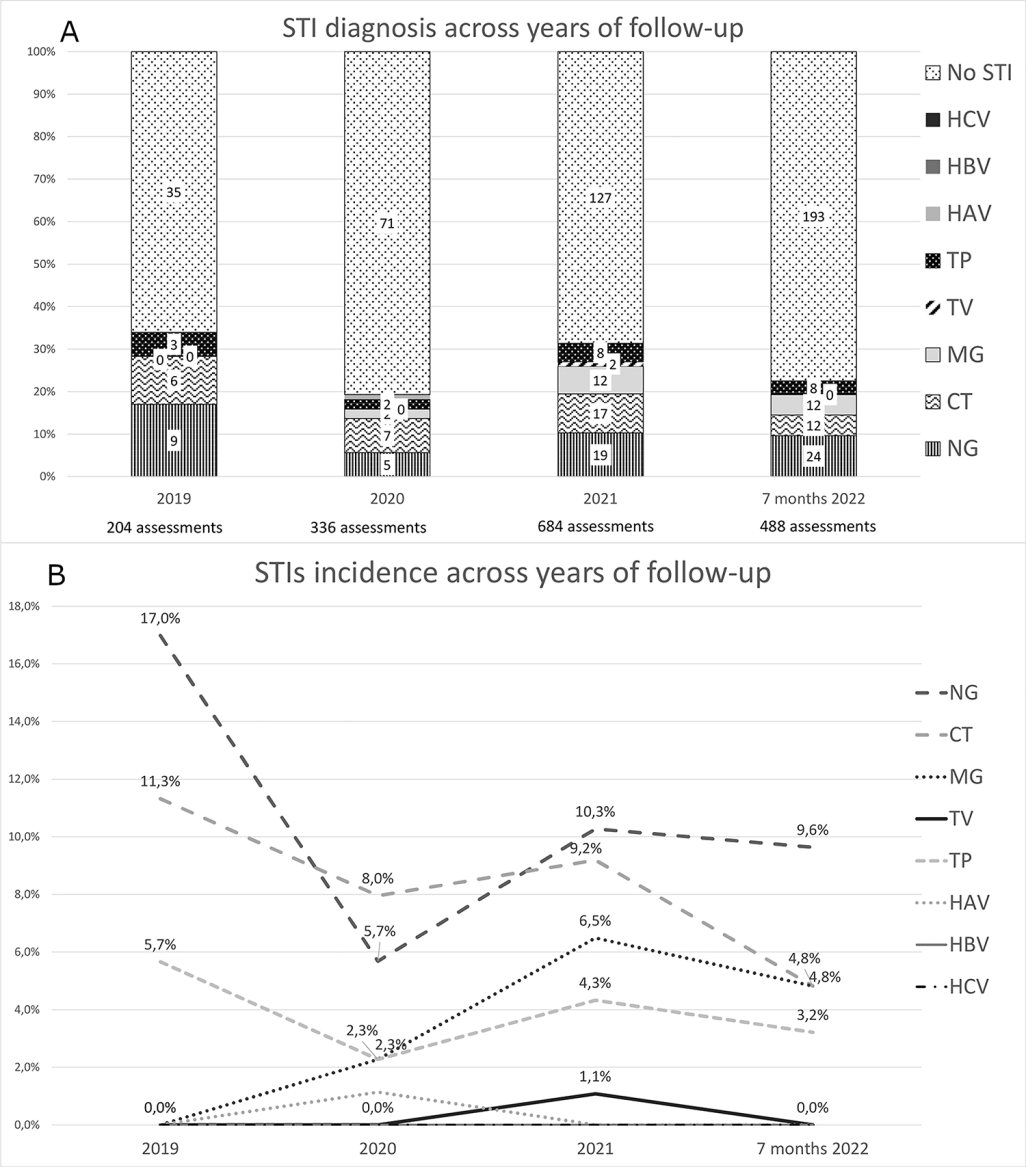
**A**, **B** STIs incidence across years of follow-up

A total of 550 assessments for STIs were obtained and collected during follow-up, for a total number of 1712 samples including plasma, urine, rectal and pharyngeal swabs. As shown in Fig. 1a/b the incidence of STIs obtained varied annually during follow-up, although the test for trend did not reveal statistical significance.

We registered after 2020 a nearly incremental trend of NG diagnoses: 17.0% cases were reported in 2019, 5.7% in 2020, 10.3% in 2021 and 9.6% in the first 7 months of 2022. MG also presented a potential positive trend, being found in 2.3% PrEP users in 2020, 6.5% in 2021 and 4.8% in the first 7 months of 2022.

On the contrary, a stable trend was found for CT incidence, from 11.3% in 2019 to 8.0% in 2020, to 9.2% in 2021 and to 4.8% in 2022.

Only two cases of TV were diagnosed in 2021, both presenting with urethritis in cisgender MSM. TP incidence was fluctuating during the follow-up: 5.7% in 2019, 2.3% in 2020, 4.3% in 2021 and 3.2% in the first 7 months of 2022.

A single case of HAV infection was detected in 2020. No cases of HBV, HCV and HIV infection were recorded during the follow-up period.

**Fig. 2:**
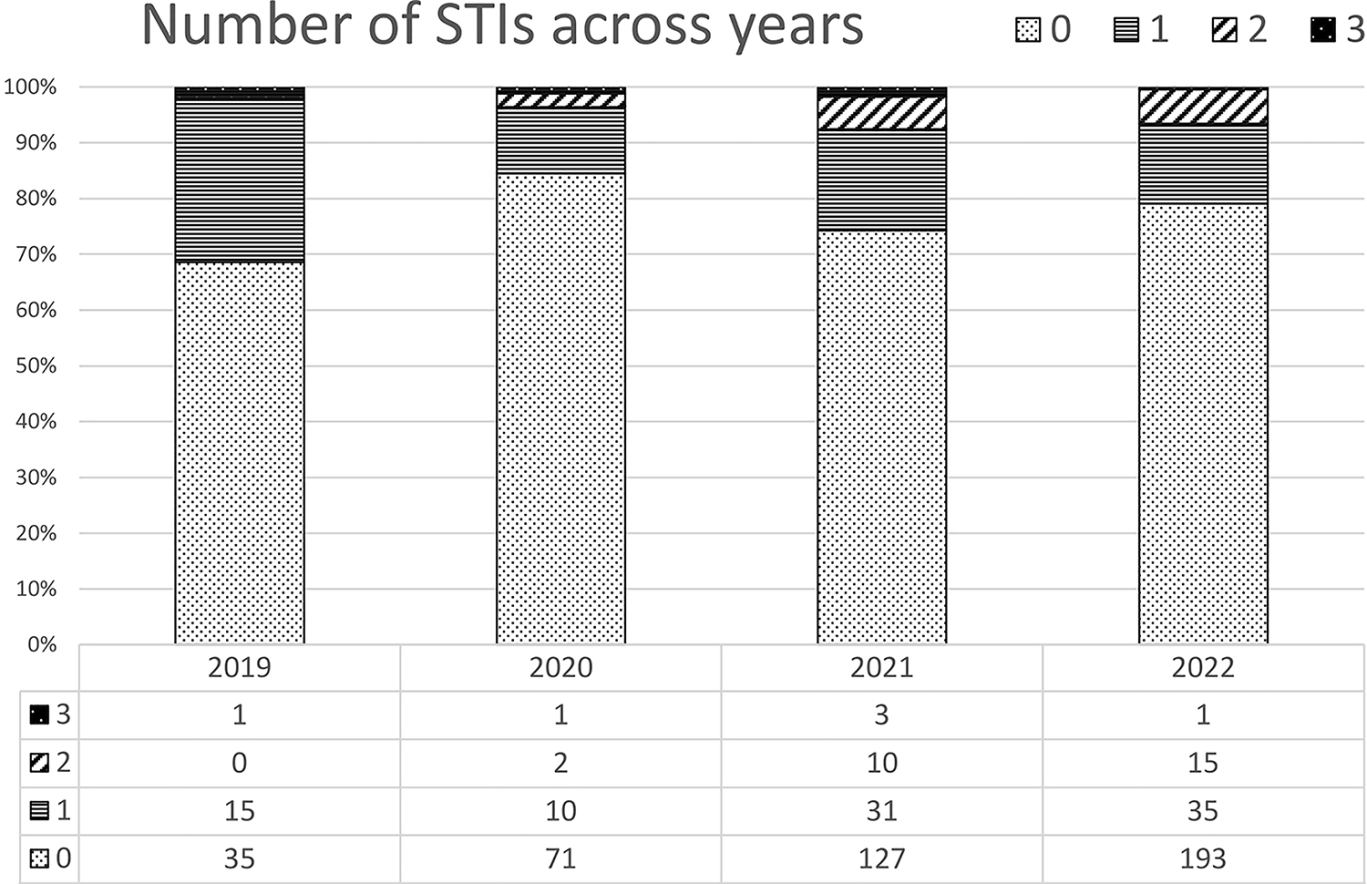
Number of individuals with single or multiple STI diagnosis across years of follow-up

The proportions of subjects with one or multiple STI diagnoses varied across years of follow-up (Fig. 2), with the highest prevalence of 1, 2 and 3 STIs in 2019, 2022 and 2019 respectively, and the lowest of 1, 2 and 3 STIs in 2020, 2019 and 2022 respectively. In particular, in 2019 the proportion of users with 1, 2 or 3 STIs were 29.4%, 0% and 2% respectively; these figures turned to 11.9%, 2.4% and 1.2% in 2020 and to 18.1%, 5.9% and 1.8% in 2021; in the first 7 months of 2022 they were 14.3%, 6.2% and 0.4%. The test for trend across year groups showed no statistical significance for all the STIs.

At logistic regression models (Table 3), the only factor that resulted associated to risk of STIs during the follow-up was history of STIs before PrEP start (OR 3.37, 95%CI 1.27–8.94, p 0.014).

**Table 3:** Logistic regression analysis: outcome risk of stis

Variable	OR	95% CI	*P* value
Sex	0.68	0.04–12.30	0.796
Italian vs. not Italian	1.13	0.25–5.24	0.873
Age at PrEP start			
< 30 ref	–	–	–
31–50	0.77	0.24–2.44	0.660
> 50	0.97	0.20–6.62	0.972
Initiation reason	0.69	0.24-2.00	0.495
Regimen type (on demand vs. daily)	1.28	0.63–2.58	0.498
History of STIs	3.37	1.27–8.94	0.014
Condom use yes vs. no	0.45	0.08–2.40	0.351

### Nephrotoxicity Sub-analysis

Complete data about serum creatinine were available for 189 out of 244 subjects (77.5%), and were included in the nephrotoxicity sub-analysis. In this subset of users, median follow-up time was 7 (IQR 3–20) months. 7 cases of nephrotoxicity were recorded according to previously mentioned definition (described in Table 4).

**Table 4:** Shows the specific nephrotoxicity data collected

	Basal creatinine level (mg/dl)	Last creatinine level (mg/dl)	Multiple imputation yes/no	Increase (mg/dl)	Age (years)	Length of follow-up (months)	PrEP regimen	PrEP Interruption
User 1	1.02	1.34	No	0.32	50	36	On-demand	No
User 2	0.71	0.86	No	0.15	41	31	On-demand	No
User 3	1.36	2.24	No	0.88	49	2	On-demand	Yes (due to nephrotoxicity)
User 4	0.77	0.96	No	0.19	39	11	Missing	No
User 5	0.82	1.00	No	0.18	31	1	On-demand	No
User 6	0.79	0.99	No	0.20	29	15	On-demand	No
User 7	0.87	1.06	No	0.19	46	3	On-demand	No
User 8	0.6	0.93	Yes	0.33	37	21	Daily	Yes (not due to side effects)
User 9	1.05	1.30	Yes	0.25	55	9	Daily	No
User 10	0.99	1.22	Yes	0.23	49	24	On-demand and daily	Yes (not due to side effects)
User 11	0.92	1.18	Yes	0.26	64	6	On-demand and daily	Yes (not due to side effects)

We observed mild variations in creatinine levels: the maximum rise was 0.88 mg/dl (from 1.36 to 2.24 mg/dl) in a single user, that was contemporary co-medicated with lisinopril due to hypertension and that was under PrEP regimen by 2 months. PrEP administration was stopped only in this case and after the interruption the creatinine level returned to baseline value. The median follow-up time in subjects who experienced nephrotoxicity was 11 (IQR 1–32) months; three out of 7 users presented nephrotoxicity within 6 months after PrEP initiation.

In the other cases PrEP was continued reinforcing hydration. Six out of 7 users were using on-demand PrEP regimen and no differences were found according to age and sex.

As explained in the methods section, a further analysis has been done after multiple imputation to address 22% of missing data regarding baseline and final creatinine values. The new analysis with imputed dataset produced a total of 11 (4.5%) subjects with nephrotoxicity (as showed in Table 3), and we obtained confirmation of the absence of differences according regimen type, age and sex.

## Discussion

Our study demonstrated the efficacy and safety of HIV prevention through TDF-based PrEP, as known from literature. Nevertheless, many concerns are still ongoing about how this preventive strategy may impact on the possibility to acquire other STIs.

Regarding demographic characteristics of our cohort, these are similar to others European and American reported in literature, being the majority of patients MSM who started PrEP due to frequent risky sexual behaviours [3, 22, 23, 24]. Notably, in our cohort the numbers of cis-gender and transgender women are roughly in line with the ones reported in other massive studies [16, 25] on this topic and this make easier to speculate about the impact of PrEP on STIs acquisition because the populations described are similar.

The majority of users were Italian in our cohort (91.8%): this data may highlight the persistent presence of a social barrier in the access to public health services, such as PrEP for some specific populations in Italy (foreign people, sex workers, etc.). Moreover, at the time of data collection, PrEP was not reimbursed by the National Health System, this means that for some people it might have not been affordable, especially for those who live in less-favourable social-economic conditions as more often reported for foreign people in our country.

Compared to other studies, in our cohort a higher percentage (15.6%) of the users started PrEP above 50 years of age. Notably, the reasons to start the prophylaxis were similar to younger users (unprotected sexual intercourses and personal safety). Interestingly, despite the potential higher risk of renal toxicity linked to the TDF use in this older sub-population, none of them developed significant toxicities, being the loss to follow up and the lockdown during pandemic the only reason given to stop assuming PrEP.

STIs have an important impact on sexual and reproductive health not only in middle/low-income countries but also in high ones. In fact, increasing STIs are associated with rise in serious illness as well as the risk of antibiotic resistance, particularly involving *NG* strains in the last decades [26]. These concerns drove the setting of many recent studies that aim to evaluate the best strategy to prevent STIs transmission, as for example the use of post-exposure antibiotic prophylaxis (PEP). Comparing to what reported by Luetkemeyer et al. [16] in their PrEP cohort not assuming Doxycycline-PEP, the prevalence of NG and CT per year in our cohort is lower for NG and CT (20.2% and 12.1% vs. 10.3% and 9.2% respectively, in 2021). Historically the prevalence of NG and CT infections in Italy is lower than the one reported in other countries in Europe (i.e. France) [3] as well as in USA or Australia [16,27, 28] where bigger studies about impact of PrEP in STIs acquisition were settled [13, 28].

However, even if a relatively small percentage of these infections is reported in our cohort, it is important to highlight the positive trend in NG infection recorded in the last year of the data collection, especially considering that it was arrested just in the middle of 2022, and that a doubling in NG infections had been already pointed out in Italy in 2019 [29]. Moreover, our sample is relatively small and our study takes place during the first 2 years of COVID-19 pandemic, during which straight social restrictions were ongoing as well as the access to PrEP follow-up clinics. For all these reasons, it is possible to suppose that the incidence of STIs in our cohort is underestimated. On this background, despite the low relative incidence reported in our cohort, we support the idea the Doxy-PEP could be a valid strategy to prevent the spread of STIs in Italy. Unfortunately, since NG infections are detected only by molecular assay and no swab culture is routinely performed, it is not possible to estimate the prevalence of strain resistant to doxycycline and so the actual efficacy of this approach in our reality.

Another aspect to consider is whether the increasing rate of STIs reported among PrEP users in large cohorts could be attributed to a “behavioral compensation”, with consequent decreased infectious risk perception, or to a better monitoring as a consequence of more frequent STI testing. This unsolved issue could justify the discrepancy between our low incidence of STIs and the one reported in other big trials. As a factor associated to risk of STIs during the follow-up at our logistic regression results, we identified only history of STIs before PrEP start.

In fact, Nguyen et al. [30] clearly retrospectively reported a higher incidence of STIs in the first year after starting PrEP and same results were confirmed by another big prospective observational study settled in Australia [26]. In both cases the authors hypothesized that the higher incidence of STIs reported in 1 year after starting PrEP may be linked with behavioral changes in PrEP users, more prone to underestimated the risk of STIs acquisition (other than HIV). Notably, we didn’t observe in our cohort significant change in STIs incidence after starting PrEP, which in any case was consistent with other reports from recent European literature [23, 24, 25]. Nevertheless, after the end of lockdown in 2021 the number of PrEP users doubled, rising from 33 to 87 (49 new users and 5 reintroduced PrEP after a stop), together with an initial trend upward of STIs incidence (both regarding the single infections and their cumulative number), in line with what reported by the last national report available monitoring STIs trend in Italy [29].

Different studies have been recently published regarding trends of PrEP adherence during COVID-19 pandemics: as in the HIV care, also the PrEP services presented disruption or difficulties on the engagement of PrEP care continuum, especially in the key populations. Thus, it’s possible to speculate that the lockdown restrictions and the relative low numbers of PrEP users (51 in 2019) all together contributed to mask the real size of STIs spreading issue in our country. Thereby, we strongly support the systematic screening in PrEP users in order to early detect and treat STIs, especially the asymptomatic one, to reduce the number of missing infections in PrEP users [18, 31].

Despite no clear recommendations are given by current guidelines about HAV vaccination in PrEP users [2], in our centers we recommend the vaccination against hepatitis A and B viruses in seronegative users. The high efficacy of this approach may be positively linked to the almost absence of new hepatitis virus infections observed in our cohort, except for a single HAV case in 2020. On these evidences, we think that HAV/HBV vaccination should be recommended as another valid option to prevent the transmissions of hepatitis viruses in PrEP users.

Regarding the nephrotoxicity of TDF in PrEP regimen, few data from literature deal with this concern, not leading to definitive conclusions. A systematic review conducted by Pilkington et al. [32] including 13 RCTs did not find evidence of correlation between TDF-based prophylaxis and renal serious adverse events, encouraging the administration to people at risk of HIV infection. Schaefer et al. [33], in their review, confirm the safety of TDF in prophylaxis regimens showing the low proportion of clinically significant renal damage and the reversibility of it after discontinuation of the drug. Moreover, they highlighted the correlation between age above 50 years, basal renal function (creatinine clearance < 90 ml/min) and the potential TDF-based regimen nephrotoxicity. Though the median age of PrEP users, across numerous studies, is around 30–35 years old, the higher proportion of ageing people (above 50 years old) in our cohort may suggest the need to take into account this population in future works. Current CDC guidelines suggest alternative prophylaxis regimens in case of known kidney disease, i.e. tenofovir alafenamide based regimen for eGFR > 30 ml/min and injectable cabotegravir based regimen for those with a severe renal disease, although specific precautions toward older patients are not indicated. At the time of our data collection TDF regimen was the only affordable choice for our users. In our cohort, even after multiple imputation analysis, we did not find a high level of toxicity, confirming the hypothesis that TDF is safe for this medical indication. Notably PrEP medication was stopped only in one patient who was contemporarily assuming a potential nephrotoxic drug. This highlights the importance of a complete collection of other risk factors for nephrotoxicity (life style, comorbidities and comedications) in order to prevent it, especially in the ageing population, known to be at higher risk of comorbidities [19, 33, 34].

Our study presented some limitations, due mainly to the retrospective design and to the lack of a comparison cohort not on PrEP: in particular this could help to understand whether a PrEP may be an actual driver of higher STI incidence by leading users to changes in their behavior. A main limitation to our study was the significant proportion of missing data regarding creatinine values: as we described above, the multiple imputation analysis helped us to mitigate the impact of missing data on our study results and uphold their credibility.

Another limitation to our study is the lack of further follow-up about patients with nephrotoxicity, in order to assess kidney function recovery for all the users.

## Conclusions

PrEP is an effective strategy for HIV transmission prevention, and nowadays is widely suggested for sexually active population. Our PrEP cohort was similar to literature, with a significant presence of users above 50 years of age. The lower access to service in some populations at risk reflects the need for an awareness campaign about prevention strategies of HIV spread, together with an effort to make them easily available and affordable. Moreover, further studies are needed to investigate the safety and efficacy in either heterosexual and transgender women.

The impact of COVID-19 pandemics on the PrEP care continuum has been described in literature and our study confirmed a deflection in PrEP adherence and in STIs testing during 2020.

STIs incidence is rising worldwide, in particular after COVID-19 pandemics lockdown restrictions, with concern regarding NG and CT. Thus, prevention, routine screening, early detection and treatment, may be useful to limit STIs spread in the PrEP population.

Our data demonstrated low level nephrotoxicity of TDF for PrEP indication, that may be considered as safe. Further studies in real world setting with longer follow-up and use of different drugs, such as Tenofovir alafenamide or long acting Cabotegravir, may investigate both the kidney function recovery and the availability of a safe alternative strategy for users who develop toxicity or for those with a higher risk of nephrotoxicity at baseline.
